# Improvements to executive function during exercise training predict maintenance of physical activity over the following year

**DOI:** 10.3389/fnhum.2014.00353

**Published:** 2014-05-27

**Authors:** John R. Best, Lindsay S. Nagamatsu, Teresa Liu-Ambrose

**Affiliations:** ^1^Department of Physical Therapy, University of British ColumbiaVancouver, BC, Canada; ^2^Djavad Mowafaghian Centre for Brain Health, University of British ColumbiaVancouver, BC, Canada; ^3^Centre for Hip Health and Mobility, University of British ColumbiaVancouver, BC, Canada; ^4^Department of Psychology, University of British ColumbiaVancouver, BC, Canada; ^5^Brain Research Centre, University of British ColumbiaVancouver, BC, Canada

**Keywords:** executive function, aging, physical activity adherence, exercise training, temporal self-regulation theory

## Abstract

Previous studies have shown that exercise training benefits cognitive, neural, and physical health markers in older adults. It is likely that these positive effects will diminish if participants return to sedentary lifestyles following training cessation. Theory posits that that the neurocognitive processes underlying self-regulation, namely executive function (EF), are important to maintaining positive health behaviors. Therefore, we examined whether better EF performance in older women would predict greater adherence to routine physical activity (PA) over 1 year following a 12-month resistance exercise training randomized controlled trial. The study sample consisted of 125 community-dwelling women aged 65–75 years old. Our primary outcome measure was self-reported PA, as measured by the Physical Activity Scale for the Elderly (PASE), assessed on a monthly basis from month 13 to month 25. Executive function was assessed using the Stroop Test at baseline (month 0) and post-training (month 12). Latent growth curve analyses showed that, on average, PA decreased during the follow-up period but at a decelerating rate. Women who made greater improvements to EF during the training period showed better adherence to PA during the 1-year follow-up period (β = −0.36, *p* < 0.05); this association was unmitigated by the addition of covariates (β = −0.44, *p* < 0.05). As expected, EF did not predict changes in PA during the training period (*p* > 0.10). Overall, these findings suggest that improving EF plays an important role in whether older women maintain higher levels of PA following exercise training and that this association is only apparent after training when environmental support for PA is low.

## Introduction

Aerobic and resistance exercise training over several months improve cognitive function (Kramer et al., 1999; Colcombe et al., 2004; Cassilhas et al., 2007; Lautenschlager et al., 2008; Liu-Ambrose et al., 2010; Nagamatsu et al., 2012), alter brain function and morphology (Colcombe et al., 2004; Liu-Ambrose et al., 2010, 2012; Voss et al., 2010; Erickson et al., 2011; Nagamatsu et al., 2012), and improve physical fitness (Colcombe et al., 2004; Erickson et al., 2011) and mobility (Nagamatsu et al., 2012) in community-dwelling older adults. It is likely, however, that these positive effects to brain and body will wane over time if older adults return to sedentary lifestyles following training cessation. Indeed, research suggests that adherence to physical activity (PA) decreases following exercise programs. One study reported that only 25% of older men and women continued with aerobic exercise 6 months following a structured 12-month aerobic walking program (Tak et al., 2012), while another showed that only 17% of older women met the walking recommendations (≥150 min per week) 21 months following a 3-month home based walking and balance program (Findorff et al., 2009). Given the decline in PA following exercise programs, it is important to understand why some older adults maintain high levels of PA, whereas others become more sedentary following training.

Temporal self-regulation theory posits that the neurocognitive processes underlying self-regulation are critical to the uptake and maintenance of health-promoting behaviors, including PA (Hall and Fong, 2007; Hall, 2013). These neurocognitive processes are commonly referred to as executive function (EF) and are underpinned by neural networks involving the prefrontal cortex (Shimamura, 2000). There are a variety of EF assessments, but one of the most famous is the Stroop task (Stroop, 1935). The key condition in this task requires individuals to identify aloud the ink color for color words whose name conflicts with the ink color (e.g., the word “blue” is presented in a red font). This condition creates interference that must be overcome because the natural tendency is to identify the word and not the color of the word. Hence, the key aspect of EF recruited on this task is the ability to override a prepotent response in favor of an alternative response. Individuals who perform better on this task (i.e., respond more quickly and/or more accurately) tend to act less impulsively and are better able to resist temptations, as determined by self- and informant-reports (Duckworth and Kern, 2011). They also tend to be more future-oriented, meaning that they discount the value of delayed outcomes *less* than their peers with poorer Stroop performance (Duckworth and Kern, 2011). Additionally, the neural systems associated with Stroop performance (e.g., lateral prefrontal cortices) have been shown to be important to modulating the value of immediate vs. delayed rewards and to avoiding immediate temptations that may have negative long-term consequences (McClure et al., 2004; Shamosh et al., 2008; Hare et al., 2009; Kober et al., 2010).

Although older adults may generally perceive PA to be valuable and health promoting, the costs and barriers associated with PA can be deterrents (Costello et al., 2011). Engaging in PA therefore may require an individual to exert significant effort and to place substantial value on the receipt of positive benefits in the future (e.g., reduced weight, better cardiovascular health) in order to endure immediate costs (e.g., discomfort associated with exertion, opportunity costs related to forgoing sedentary activities) and to overcome barriers (e.g., limited leisure time, lack of nearby recreational facilities). Thus, compared to individuals with lower EF, individuals with higher levels of EF may be more likely to engage in PA routinely because they are better capable of overriding impulses for immediate rewards and of shifting focus to delayed outcomes. This hypothesis follows from the previously mentioned findings that better EF performance is associated with higher levels of self-regulation and to greater future orientation. In thinking about exercise training programs, this association between EF and PA should be especially evident once the structured exercise training has ended, and external support (e.g., encouragement from trainers, incentives for participation) for PA is diminished. At this point, the barriers and costs related to PA increase, and the influence of internal motivational and self-regulatory processes on decisions to engage in PA will likely be important.

The current study is a secondary analysis of a randomized controlled trial (RCT) in which healthy, community-dwelling women (aged 65–75) were randomized to a once-weekly resistance training, twice-weekly resistance training, or twice-weekly balance and tone training, each of which had duration of 52 weeks. We have previously shown that both frequencies of resistance training had a positive impact on executive function, as assessed by change in performance on the Stroop test from baseline to post-training, in comparison to balance and toning (Liu-Ambrose et al., 2010). The current study adds to this previous analysis by examining changes in PA levels over the year following the training program. Using monthly self-reports of PA, the first aim was to characterize the nature of change in PA over the follow-up period. The second and more important aim was to determine whether baseline Stroop performance and/or improvements in Stroop performance over the training period predicted individual variation in PA change over the follow-up period. Beyond the role of EF in temporal self-regulation theory, there are other reasons to examine EF performance in this sample. First, previous research has shown that EF is one domain of cognition sensitive to the positive effects of exercise training (Colcombe and Kramer, 2003; Smith et al., 2010). Second, EF performance has been shown to decline with older age (Jurado and Rosselli, 2007), but within a healthy community-dwelling sample of older women, there is likely substantial variation in EF performance. It was hypothesized that individuals with better performing EF, either at baseline or through improvements acquired during training, would better maintain regular PA over the subsequent year.

## Materials and methods

The 52-week training program occurred between May 1, 2007 and April 30, 2008. Details on the participants, study design, and intervention can be found elsewhere (Liu-Ambrose et al., 2010); thus, their presentation is abbreviated below. Ethical approval was obtained from the Vancouver Coastal Health Research Institute and the University of British Columbia's Clinical Research Ethics Board. The RCT was registered at clinicaltrials.gov (Identifier: NCT00426881). Assessors were blinded to the participants' exercise training assignment.

### Participants

We recruited and randomized 155 women aged 65–75, who lived in Vancouver, British Columbia. All participants lived independently in the community, had intact cognitive functioning and acceptable visual acuity with or without corrective lenses, and spoke English fluently. Potential participants were excluded if they had a medical condition contraindicating physical exercise, had participated in resistance training in the past 6 months, had a neurodegenerative disease, stroke, or depression, or were receiving estrogen or testosterone therapy. All participants provided written informed consent.

### Exercise interventions

Resistance training and balance and tone classes were led by certified fitness instructors. Classes were 60 min long, with a 10-min warm-up and 10-min cool-down. The resistance training implemented a progressive, high intensity protocol using Keiser pressurized air system and free weights. Non machine- or weight-based exercises included mini-squats, mini-lunges, and lunge walks. The balance and toning program used stretching, range-of-motion, core-strength, balance, and relaxation exercises. Only body-weight was used, with no additional loading. This group served as a control to factors secondary to study participation, such as dedication to an exercise program and socialization, by matching the twice-weekly resistance training condition for in-person contact.

### Measures

#### Executive function

The Stroop Test has three conditions, each containing 80 items. First, participants read out loud color words printed in black ink. Second, participants read out loud the color of colored x's. Finally, participants read out loud the ink color for incongruent color words (e.g., the word “red” is printed in blue ink). The ability to selectively attend to one stimulus property while resolving conflict from competing stimulus properties is calculated by subtracting the amount of time it takes to complete the second condition from the third condition. A smaller time difference indicates better EF, and a greater decrease in the time difference from baseline to post-training indicates greater improvements to EF.

#### Physical activity (PA)

Self-reported PA was assessed using the Physical Activity for the Elderly (PASE) (Washburn et al., 1993). The PASE consists of 19 items that inquire about different categories of leisure PA. (Note: The PASE also contains 2 items regarding sedentary activity that are not scored). Participants first report the number of days per week the activity was performed over the past 7 days and then the number of hours per day. A summary PA score is computed from weights and frequency values for each of the PA categories. The psychometric properties of the PASE were determined in a sample of 277 adults aged 65–100 years (Washburn et al., 1993). In subsequent studies, PASE scores have been shown to correlate (*r* = 0.49) with objectively measured PA over 3 days via accelerometers (Washburn and Ficker, 1999) and to correlate modestly (|*r*| = 0.18 − 0.20) with objective measures of balance, peak oxygen uptake, and systolic blood pressure (Washburn et al., 1999). In the current study, PASE assessments were conducted in person at baseline (month 0), at training completion (month 12), and at the 1-year follow-up (month 25). During the follow-up period, 13 follow-up PASE assessments were conducted by mail from May 2008 and May 2009. For the final PASE assessment (month 25), the in-person assessment was used unless the participant only completed a final assessment by mail (*n* = 6). The intra-class correlation coefficient, calculated on a subset of the participants (*n* = 111) who had complete PASE data at months 0, 6, 12, and 25, was 0.81, indicating acceptable inter-temporal reliability in the PASE measure across the entire study period.

#### Covariate variables

Age in years and education level were assessed at baseline by self-report. For education, participants selected the appropriate category, which ranged from “No High School” to “University Degree.” The remaining covariates were assessed at baseline and upon training completion (month 12). Depression was assessed by the 15-item Geriatric Depression Scale (GDS) (Yesavage et al., 1982). Weight was assessed in kilograms based on the average of two measurements using a digital scale. Global cognition was assessed using the Montreal Cognitive Assessment (MoCA), which is a 10-min cognitive screening tool containing items related to language, orientation, short-term memory and EF (Nasreddine et al., 2005). The MoCA has a maximum score of 30 points, and scores ≥26 indicate intact cognition (Nasreddine et al., 2005). The number of comorbid conditions related to physical functioning was determined using the Functional Comorbidity Index (FCI) (Groll et al., 2005).

### Statistical analyses

Preliminary descriptive statistics, reliability analyses, and logistic regressions were conducted using SPSS version 21 (IBM Corporation, 2012). The main analyses to examine predictors of PA maintenance over the 1-year follow-up were conducted in Mplus version 7.11 (Muthén and Muthén, 2012) using latent growth curve models. For all models, missing data were handled by maximum likelihood estimation under the assumption that missing values were missing at random. To guard against departures from distribution normality, we ran analyses with robust standard errors. We tested model fit by the χ^2^ test, comparative fit index (CFI) and the root mean square error of approximation (RMSEA). Good fit is indicated by a non-significant χ^2^ test, CFI > 0.95, and RMSEA < 0.05 (Hu and Bentler, 1999).

The first set of growth curve models determined the best-fitting unconditional model (i.e., a model without any predictor variables). The slope factor loading for the initial PASE follow-up assessment (i.e., month 13) was fixed to zero and increased by 0.25 units for each subsequent month, resulting in the final PASE assessment being fixed at 3. By fixing the factor loadings in this fashion, the latent intercept represents the PASE score at month 13, and the slope value represents the amount of change in the PASE score that occurred every 4 months during the follow-up period. Once a satisfactory model was determined, two conditional growth models (i.e., models with predictors) were tested. The first was a base model, which added baseline Stroop performance and the change in Stroop performance over the 12-month intervention as predictors. The second was a covariate-adjusted model, which added additional predictors to determine whether any predictive effects of Stroop performance could be explained by demographic, physical, or psychological variables. Standardize path coefficients (β) are reported for the conditional models.

## Results

### Sample characteristics

Of the original 155 women who were randomized in one of the three treatment conditions at baseline, 125 (81%) completed ≥2 follow-up PASE assessments and were included in the current study. Logistic regression analyses showed that the participants included in this study did not differ from those who were excluded on baseline demographic or other variables of interest (all *p*s > 0.08). Of the current sample, 57 women (46%) completed the PASE at all follow-up time points, and the rate of completion at any given follow-up time point ranged from 70 to 97%. In a preliminary analysis, we determined that Stroop performance (baseline and change during treatment) did not predict the number of PASE assessments completed during the follow-up period (*p*s > 0.55). Table 1 provides descriptive statistics for the predictor variables for the study sample. Of note, the average baseline Stroop interference score was 46.30 s (*SD* = 19.27 s), and the average reduction in Stroop interference score from baseline to month 12 was 4.75 s (*SD* = 18.38 s), which represents a significant improvement, *t*_(123)_ = −2.81, *p* < 0.01. Table 2 shows the bivariate correlations among these variables of interest. Notably, women with better baseline Stroop performance had higher baseline global cognition as assessed by the MoCA (*r* = −0.31). Heavier women at baseline and women with better baseline Stroop performance showed smaller improvements to Stroop performance over the course of the intervention (*r* = 0.18 and −0.64, respectively).

**Table 1:** **Descriptive statistics for predictor and covariate variables for the study sample and by treatment condition**.

		**Treatment condition**		
**Variable**	**Total (N = 125)**	**BAT (n = 36)**	**1 × RT (n = 44)**	**2 × RT (n = 45)**
Age (years)	69.54 (2.85)	69.81 (2.92)	69.46 (2.61)	69.42 (3.01)
**EDUCATION, NO. (%)**				
No high school	1 (0.8)	0 (0.0)	1 (2.3)	0 (0.0)
Some high school	8 (6.4)	2 (5.6)	2 (4.5)	4 (8.9)
Complete high school	20 (16.0)	5 (13.9)	7 (15.9)	8 (17.8)
Trade or professional	23 (18.4)	10 (27.8)	10 (22.7)	3 (6.7)
University certificate	23 (18.4)	5 (13.9)	9 (20.5)	9 (20.0)
University degree	50 (40.0)	14 (38.9)	15 (34.1)	21 (46.7)
PASE, bl	123.57 (59.75)	130.83 (52.33)	117.64 (62.92)	123.57 (61.47)
PASE, Δ	−0.61 (59.50)	−4.43 (57.94)	4.62 (66.91)	−1.49 (51.43)
GDS, bl	0.49 (1.73)	0.22 (1.31)	0.27 (1.03)	0.91 (2.37)
GDS, Δ	0.15 (1.44)	0.38 (1.24)	0.13 (1.04)	0.00 (1.85)
FCI, bl	2.08 (1.61)	2.17 (1.64)	1.84 (1.66)	2.24 (1.51)
FCI, Δ	−0.28 (1.34)	−0.07 (1.24)	−0.34 (1.44)	−0.44 (1.26)
MoCA (max. 30 pts), bl	25.11 (3.00)	25.36 (3.12)	24.73 (3.03)	25.29 (2.83)
MoCA, Δ	−0.31 (3.21)	0.03 (3.26)	0.11 (3.11)	−1.00 (3.15)
Weight, bl (kg)	70.54 (13.83)	69.71 (10.36)	71.45 (15.48)	70.31 (14.47)
Weight, Δ (kg)	−0.34 (3.55)	−1.03 (4.44)	−0.26 (2.83)	0.08 (3.23)
Stroop, bl (s)	46.30 (19.27)	46.36 (14.85)	46.72 (24.72)	46.08 (16.02)
Stroop, Δ (s)	−4.75 (18.38)	−1.98 (17.01)	−6.39 (22.76)	−5.27 (13.70)

Δ*, 12-month score minus baseline score; 1 × RT, weekly resistance training condition; 2 × RT, twice weekly resistance training condition; BAT, balance and toning condition; BL, baseline; FCI, functional comorbidity index; GDS, geriatric depression scale; MoCA, montreal cognitive assessment; PASE, physical activity scale for the elderly; Stroop, Stroop interference score measured in seconds*.

**Table 2:** **Bivariate correlations among predictor and covariate variables**.

**Variable**	**1.**	**2.**	**3.**	**4.**	**5.**	**6.**	**7.**	**8.**	**9.**	**10.**	**11.**	**12.**	**13.**	**14.**
1. Age	–	**−0.21**	−0.14	−0.03	0.08	−0.02	0.15	−0.07	0.01	−0.16	0.02	−0.01	0.07	0.06
2. Education		–	0.14	−0.01	−0.03	0.04	0.03	0.02	**0.26**	−0.07	0.01	−0.01	−0.11	−0.07
3. PASE, bl			–	**−0.50**	−0.11	0.06	−0.11	−0.03	0.14	−0.16	**−0.22**	−0.12	−0.06	0.03
4. PASE, Δ				–	0.06	0.05	−0.10	0.12	0.03	0.06	0.02	0.11	0.01	−0.08
5. GDS, bl					–	**−0.54**	0.08	0.16	−0.07	0.08	**0.25**	0.13	0.03	−0.01
6. GDS, Δ						–	0.07	0.06	0.03	−0.05	0.02	−0.04	0.01	−0.02
7. FCI, bl							–	**−0.49**	0.06	−0.17	**0.25**	−0.01	−0.01	0.14
8. FCI, Δ								–	−0.08	0.15	**−0.22**	0.14	0.16	−0.22
9. MoCA, bl									–	**−0.54**	0.13	−0.01	**−0.31**	0.16
10. MoCA, Δ										–	−0.08	0.04	0.14	−0.13
11. Weight, bl											–	−0.07	−0.04	**0.18**
12. Weight, Δ												–	0.03	0.04
13. Stroop, bl													–	**−0.64**
14. Stroop, Δ														–

*N = 125. Pearson product-moment correlations are presented except those involving education, which are Spearman's rho. Significant correlations are noted in bold font, p < 0.05*. Δ*, 12-month score minus baseline score; BL, baseline; FCI, functional comorbidity index; GDS, geriatric depression scale; MoCA, montreal cognitive assessment; PASE, physical activity scale for the elderly; Stroop, stroop interference score measured in seconds*.>

PASE scores decreased steadily from month 13 through month 20 (corresponding to the month of December) and then increased through the spring months (see Table 3). Three extreme PASE values were identified (495.10, 842.53, and 908.89) from three distinct individuals that were outliers (>3 *SD*s above mean) both relative to the scores of the sample at that time point and relative to the individual's scores at other time points. These values were removed from the estimation of the growth models.

**Table 3:** **Means (standard deviations) and number of assessment completers for PASE scores over the 1-year follow-up**.

**Month 13**	**Month 14**	**Month 15**	**Month 16**	**Month 17**	**Month 18**	**Month 19**
143.37 (60.42)	134.44 (62.36)	133.45 (62.87)	133.87 (62.43)	137.61 (68.34)	128.50 (65.31)	122.10 (55.38)
*n* = 105	*n* = 102	*n* = 105	*n* = 105	*n* = 100	*n* = 101	*n* = 100
**Month 20**	**Month 21**	**Month 22**	**Month 23**	**Month 24**	**Month 25**	
101.15 (55.26)	113.62 (54.91)	122.55 (55.96)	120.38 (54.79)	125.68 (54.24)	123.78 (58.94)	
*n* = 99	*n* = 91	*n* = 88	*n* = 90	*n* = 94	*n* = 114	

### Unconditional growth models

**Figure 1:**
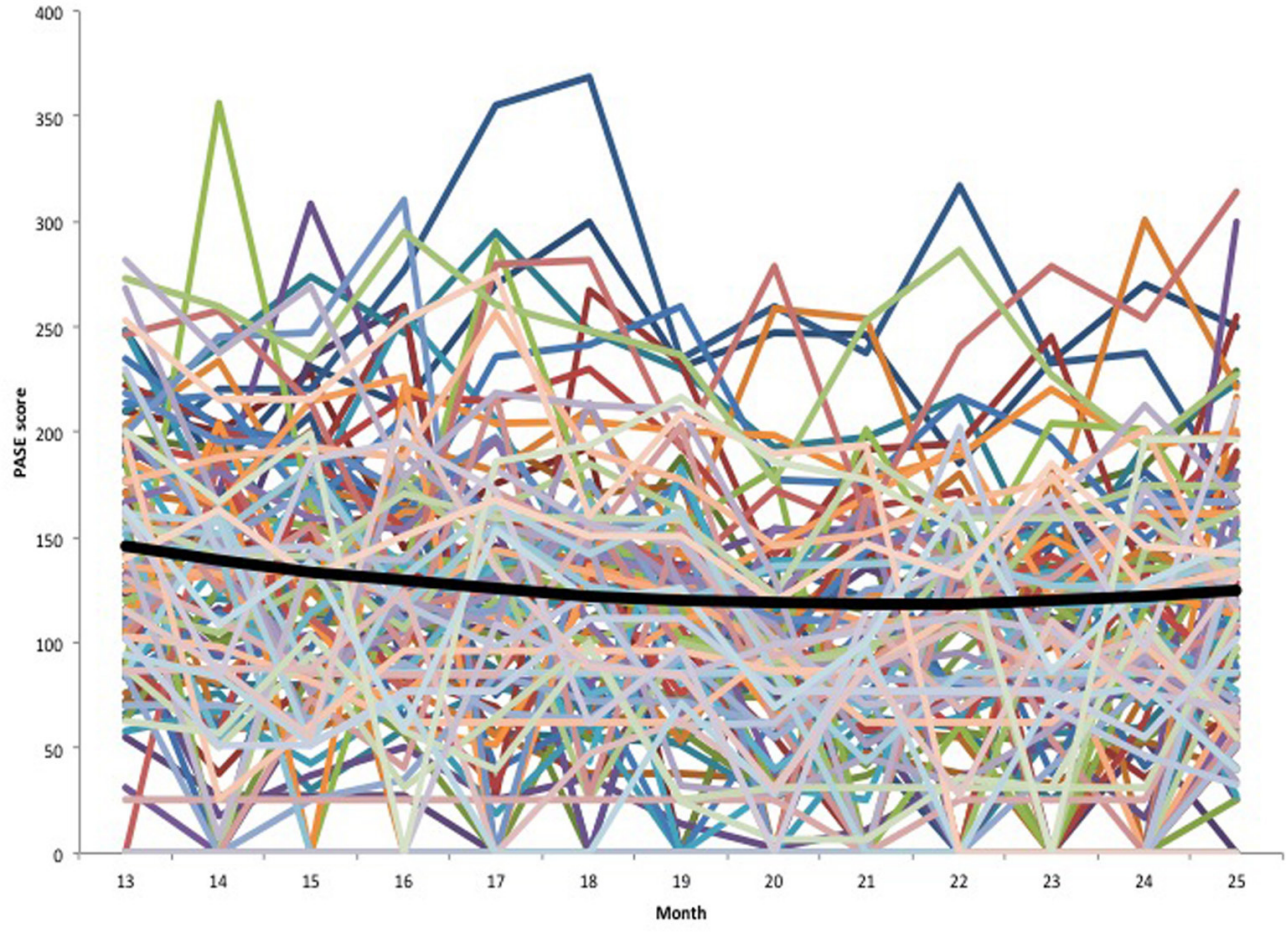
**Unconditional growth model**. Individual growth trajectories are indicated by the colored lines. The average growth trajectory specified by Model C in Table 4 is indicated by the thicker black line.

Three variations of the unconditional growth model were considered to characterize the change in the PASE follow-up scores over time (see Table 4). A model with both linear (i.e., time) and quadratic (i.e., time^2^) slope factors (model B) provided better fit than a model with only a linear slope factor (model A); however, in this quadratic model, the variances of the slope factors were not significant. Because the linear slope variance had been significant in model A, the variance for the higher-order quadratic slope was fixed to zero to produce model C, which resulted in significant variance in the linear slope. This model was chosen as the basis for the main analyses due to its superior model fit statistics compared to models A and B (Duncan et al., 2006; Muthen et al., 2011)^1^. The negative linear slope (*B* = −26.88) indicates that PA decreased over time; however, the positive quadratic slope (*B* = 6.60) indicates that this negative trend decelerated and reversed course before the end of follow-up year. Figure 1 displays each individual PASE trajectory (colored lines) along with the average trajectory (black line) specified by model C. The figure shows the substantial variation in PASE scores across time points. It also shows the quadratic effect—the black line begins to bend upward during the second-half of the follow-up period.

**Table 4:** **Determination of best-fitting unconditional growth curve model**.

	**Fit statistics**				**Intercept**		**Linear slope (Time)**		**Quadratic slope (Time^2^)**	
**Model**	**χ^2^ (*df*)**	**BIC**	**RMSEA**	**CFI**	**Mean**	**Var.**	**Mean**	**Var.**	**Mean**	**Var.**
A. Linear	122.98^**^ (86)	13421.94	0.059	0.945	136.49^***^	2537.99^***^	−6.53^***^	85.76^*^	–	–
B. Quadratic	101.85 (82)	13414.36	0.045	0.971	145.70^***^	2198.22^***^	−26.80^***^	132.45	6.54^***^	17.13
C. Quadratic	104.09 (85)	13403.05	0.043	0.972	145.62^***^	2513.89^***^	−26.88^***^	85.26^*^	6.60^***^	0.00

*df, degrees of freedom; BIC, bayesian information criteria; CFI, comparative fit index; RMSEA, root mean square error of approximation; Var., variance*.

* *p < 0.05*,

** *p < 0.01*,

*** *p < 0.001*.

### Conditional growth models

**Figure 2:**
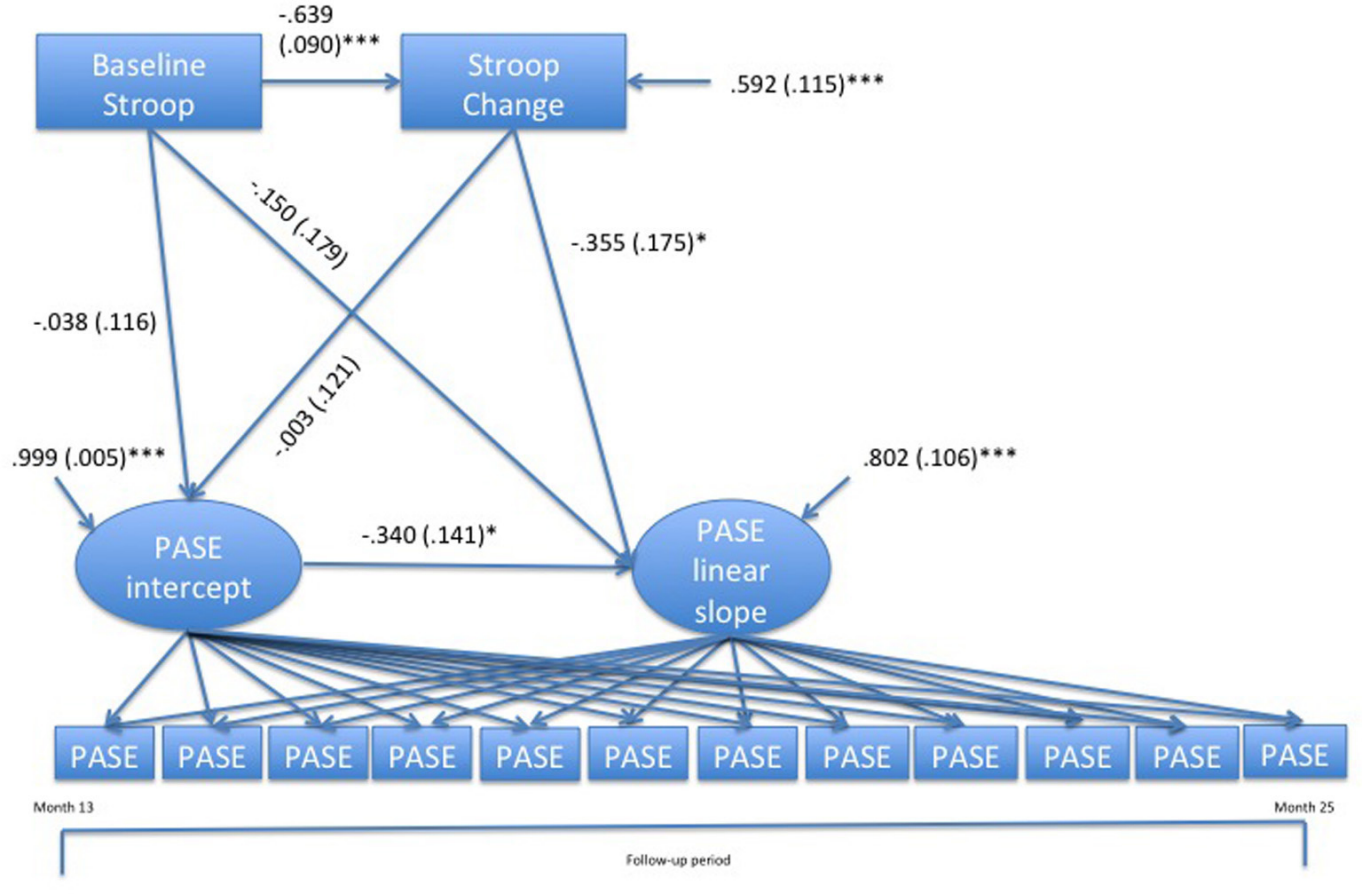
**Conditional growth curve model of the predictive effects of change in Stroop performance during the intervention on physical activity during the 1-year follow-up period**. Standardized path coefficients (standard errors) and standardized residual variances (standard errors) are shown. To simplify the depiction of the model, the PASE quadratic slope factor, the slope and intercept factor loadings, and the residual variances for PASE indicator variables have been omitted. ^*^p < 0.05. ^***^p < 0.001.

Because the quadratic slope variance was fixed to zero (indicating no heterogeneity in the rate of deceleration), the conditional growth curve models could only examine whether the baseline and change variables predicted individual differences in the PASE intercept and linear slope. The base conditional growth model (see left-hand columns of Table 5) had good fit based on a non-significant χ^2^, RMSEA < 0.05, and CFI > 0.95. In this model, women who showed greater improvements in Stroop performance reported greater maintenance of PA over the subsequent one-year follow-up period (β = −0.36, *p* < 0.05, see Figure 2). This model explained nearly 20% of the variance in the PASE slope but essentially none of the PASE intercept variance. An adjusted model was created to determine whether this predictive effect held when accounting for several important covariates theoretically related to EF and PA maintenance. These included baseline age and education, treatment condition, and baseline and change scores for the following variables: FCI, PASE, MoCA, GDS, and weight. In this adjusted model, the predictive effect of Stroop interference change on the PASE linear slope remained significant (β = −0.44, *p* < 0.05, right-hand columns of Table 5). Additionally, greater baseline PASE scores and greater increases in PASE scores over the intervention period predicted higher PASE intercepts (β = 0.89 and 0.68, respectively, *p*s < 0.001), and higher baseline global cognition (as indicated by higher MoCA scores) predicted lower PASE intercepts (β = 0.28, *p* = 0.001). Moreover, higher baseline depression and greater increases in depression over the intervention predicted greater decreases in the PASE linear slope (β = −0.50, *p* = 0.01 and β= −0.32, *p* < 0.05, respectively). This covariate-adjusted model explained over 75% of the variance in the PASE intercept and 50% of the variance in the PASE slope.

**Table 5:** **Conditional growth curve models predicting PASE intercept and slope over 1-year follow-up period**.

	**Base model**				**Covariate-adjusted model^a^**			
	**Outcome: PASE intercept**		**Outcome: PASE linear slope**		**Outcome: PASE intercept**		**Outcome: PASE linear slope**	
	**β**	***p* value**	**β**	***p* value**	**β**	***p* value**	**β**	***p* value**
**PREDICTOR**								
PASE intercept	–	–	−0.340^*^	0.016	–	–	−0.102	0.815
Stroop, baseline	−0.038	0.744	−0.150	0.401	0.053	0.493	−0.237	0.275
Stroop, Δ	−0.003	0.983	−0.355^*^	0.043	0.145	0.101	−0.441^*^	0.033
**Explained variance**	***R*^2^ = 0.001**		***R*^2^ = 0.198**		***R*^2^ = 0.756**		***R*^2^ = 0.503**	
**MODEL FIT INDICES**								
Chi−square test	χ^2^(107) = 131.90, *p* = 0.052				χ^2^(323) = 451.72, *p* < 0.01			
RMSEA (90% CI)	0.043 (0.000, 0.066)				0.057 (0.044, 0.069)			
CFI	0.970				0.900			
BIC	14,440.945				17,843.098			

*Maximum likelihood estimation with robust standard errors, N = 125. Standardized estimates (β) are presented. Δ, 12-month score minus baseline score; BIC, bayesian information criteria; CFI, comparative fit index; RMSEA, root mean square error of approximation*.

* *p < 0.05*.

a *In addition to the variables listed, this model also includes baseline age and education, treatment condition, and baseline and change scores for the following variables: Functional Comorbidity Index, Geriatric Depression Scale, Montreal Cognitive Assessment, Weight (kgs), and Physical Activity Scale for the Elderly*.

In light of the fact that we had previously found that both resistance training conditions benefited Stroop performance in this sample (Liu-Ambrose et al., 2010), we tested whether there was a significant indirect effect of resistance training on PA adherence through changes in Stroop performance. Using the model indirect command in Mplus 7.11 and bootstrapped 95% confidence intervals (5000 bootstrap resamples), we found no evidence for an indirect effect, either for once-weekly resistance training (β = 0.04 [−0.05, 0.13]) or twice-weekly resistance training (β = 0.03 [−0.06, 0.13]). The possibility that Stroop performance mediated the effects of resistance training on PA adherence was further undermined by the fact that neither once-weekly nor twice-weekly resistance training, in comparison to BAT, predicted PA adherence over the follow-up period (*p*s > 0.70).

Finally, we tested the hypothesis that the association between EF and PA adherence during the resistance-training period would be weak, perhaps because external support for PA would overshadow the influence of internal self-regulatory processes on PA. Path analysis revealed that the sign of the coefficients were negative (as expected), but neither baseline Stroop performance (β = −0.04, *p* = 0.56) nor change in Stroop performance (β = −0.13, *p* = 0.16) predicted change in PA during the training period. Furthermore, neither baseline nor change in Stroop performance predicted the number of classes attended during the 12-month training phase (*p*s > 0.40).

## Discussion

Motivated by temporal self-regulation theory (Hall, 2013), the current study tested whether individual differences in EF influence adherence to PA after completing a structured year-long exercise training program (either resistance training or balance and toning). Our results show that individuals who made greater improvements in EF during the training period showed better adherence to PA over the following year. Importantly, these findings could not be accounted for by changes in other variables, including global cognition, PA during the training period, functional comorbidity, or depression. This suggests a specific role for a key aspect of EF—namely inhibition of prepotent responses—in maintaining health-promoting behavior.

Previous studies have demonstrated the utility of temporal self-regulation theory in explaining individual differences in health outcomes in various populations (Hall and Fong, 2010). For example, Hall et al. (2006) found that better Stroop performance was related cross-sectionally to lower smoking rates, less alcohol consumption, and to better sleep quality in a community sample spanning 20–100 years of age. In another study, Hall et al. (2010) found that higher EF performance predicted a greater likelihood of survival over a 10-year period among community-dwelling older adults suffering from one or more chronic illnesses. Finally, it has been shown that overweight and obese children with a greater valuation of delayed monetary and food rewards, relative to immediate rewards, show improved weight loss (Best et al., 2012). Together, these studies suggest that exerting top-down control and valuing delayed outcomes are important predictors of positive health behaviors and outcomes.

Based on past studies alone, in which EF was only assessed at a single time point, one could not rule out that the researchers were assessing a stable individual factor that could not be altered. Thus, the most interesting contribution of the current study to temporal self-regulation theory is to show that improvements to EF predict positive health behavior adherence. Naturally, the question turns to what sorts of intervention strategies can be used to further improve EF, beyond the resistance and aerobic exercise training strategies shown to be effective in aging populations. Cognitive training has received substantial attention in recent years, and there is some evidence that training programs focused on various cognitive domains improve the targeted domain and may transfer to novel tasks in older adults (Ball et al., 2002; Willis et al., 2006; Anguera et al., 2013). There is intriguing, though tentative, evidence that training specific EF components (e.g., working memory, inhibitory control) may be especially useful in the context of improving health behavior. For example, working memory training has been shown to reduce alcohol consumption in problem drinkers (Houben et al., 2011), and inhibitory control training has been shown to reduce chocolate consumption among chocolate lovers (Houben and Jansen, 2011). Another strategy involves training individuals to engage in mental time travel in order to enhance the vividness of future outcomes; this strategy has been shown to reduce *ad libitum* energy intake in a laboratory setting (Daniel et al., 2013). Whether such strategies, either EF training or mental time travel, could improve health behavior in older adults is unknown.

It is noteworthy that change in EF, but not baseline EF, predicted PA adherence. Temporal self-regulation theory would suggest that both should be important predictors. It may be that change in EF was more sensitive in the current study because of the closer temporal proximity with the assessment of PA adherence. That is, the change in EF may provide a more accurate reflection of each person's EF performance at the time of training cessation than would the baseline assessment. It is also possible that the improvements in EF we observed may partially reflect changes in other processes during the intervention that may also be important to PA adherence. This seems unlikely given that changes in EF did not correlate with changes in any other variable studied, including change in reported PA during the intervention period (see Table 2), and that the predictive effects were unaltered in the covariate-adjusted model.

Another important contribution to temporal self-regulation theory is our result that the relationship between EF and PA adherence was not apparent during the training phase of the study. This null effect was expected because during this phase external support for PA was substantial. As examples, participants received newsletters featuring personal accomplishments, follow-up contacts when missing 2 consecutive classes without a reason, and support for overcoming barriers to participation. It is likely that the external environment modulates the influence of internal neurocognitive processes on health outcomes (Hall and Fong, 2007; Best et al., 2012; Hall, 2013), which points to additional strategies to promote health behavior. Although resource constraints would likely prevent exercise training programs to continue indefinitely, a gradual reduction in support from exercise trainers and other staff may help individuals adhere to PA. Periodic booster sessions may also help by ensuring that older adults are receiving help in overcoming barriers to PA. As our results suggest, this external support may be especially important for those individuals who do not make significant improvements in EF during the formal training period.

We also found that higher levels of baseline depression, as well as increases in depression over the training period, predicted greater decreases in PA over the follow-up period. This finding comports with the conclusion of a systematic review finding that depression is a significant risk factor for subsequent diminished PA (Roshanaei-Moghaddam et al., 2009). As the authors of that study note, depressed individuals may have lower motivation and energy levels, thus leading to a preference for sedentary activity. Together with our main results, this suggests important and distinct roles of emotional and motivational functioning, on the one hand, and self-regulatory functioning, on the other hand, in determining changes in PA over time.

There are noteworthy limitations to this study. Our study sample was fairly homogenous, as it consisted exclusively of independent community-dwelling older women who were free of major physical or cognitive impairments. This limits the generalizability of these findings to other populations. Another limitation is that we used a self-report measure of PA rather than an objective, accelerometry-based measure. Thus, our measures may be affected by many of the issues and biases associated with self-report, including cognitive limitations (e.g., inaccurate recall) and social desirability. However, by including numerous assessments (*n* = 13) of PA over the follow-up period, we believe that these data provide an accurate picture of how PA changed over that period, even in light of these issues related to self-report.

To conclude, our findings suggest an important role of cognitive plasticity during aging by showing that older women who make greater improvements to EF during an exercise training program (either resistance training or balance and toning) are more likely to adhere to PA over the following year. Our results also suggest a moderating role of environmental support, and therefore, highlight the importance of considering external environmental factors in conjunction with internal self-regulatory processes when determining optimal strategies for promoting positive health behavior.

### Conflict of interest statement

The authors declare that the research was conducted in the absence of any commercial or financial relationships that could be construed as a potential conflict of interest.

## Footnotes

^1^In secondary analyses, we determined that using model A as the basis for the conditional growth curve models provided very similar results to those reported in the text using model C. In both the base and covariate-adjusted models, change in Stroop performance predicted maintenance of physical activity over the 1-year follow-up period, β = −0.35, *p* = 0.046 and β = −0.44, *p* = 0.036, respectively.
